# Selection and Characterization of Single-Domain Antibodies for Detection of Lassa Nucleoprotein

**DOI:** 10.3390/antib9040071

**Published:** 2020-12-17

**Authors:** George P. Anderson, Jinny L. Liu, Lisa C. Shriver-Lake, Ellen R. Goldman

**Affiliations:** Naval Research Laboratory, Center for Biomolecular Science and Engineering, Washington, DC 20375, USA; george.anderson@nrl.navy.mil (G.P.A.); jinny.liu@nrl.navy.mil (J.L.L.); lisa.shriverlake@nrl.navy.mil (L.C.S.-L.)

**Keywords:** single-domain antibody, Lassa virus, Arenaviridae family, MagPlex

## Abstract

Lassa virus is the etiologic agent of Lassa fever, an acute and often fatal illness endemic to West Africa. It is important to develop new reagents applicable either for the specific diagnosis or as improved therapeutics for the treatment of Lassa fever. Here, we describe the development and initial testing of llama-derived single-domain antibodies that are specific for the Lassa virus nucleoprotein. Four sequence families based on complementarity-determining region (CDR) homology were identified by phage-based enzyme-linked immunosorbent assays, however, the highest affinity clones all belonged to the same sequence family which possess a second disulfide bond between Framework 2 and CDR3. The affinity and thermal stability were evaluated for each clone. A MagPlex-based homogeneous sandwich immunoassay for Lassa virus-like particles was also demonstrated to show their potential for further development as diagnostic reagents.

## 1. Introduction

Lassa fever, a hemorrhagic fever caused by Lassa virus (LASV), is endemic in West Africa [1]. There are numerous febrile illnesses common in West Africa, which makes Lassa fever diagnosis based solely on clinical symptoms impossible. Thus, laboratory diagnostics play a critical role in stemming the spread of this serious disease and thereby hopefully quenching an epidemic at its earliest stage. Additionally, diagnosis of Lassa fever is critical for limiting nosocomial infections, especially in the maternity ward where prospective mothers and their unborn children are at an extremely high risk of death if infected [2,3].

Several factors have served to limit the impact of Lassa fever diagnostics. Many Lassa fever patients are asymptomatic or have nonspecific symptoms [1]. This necessitates the testing of nearly all patients, creating a strain on healthcare costs. This is compounded by the fact that developing a reliable assay is complicated by the extent of LASV sequence diversity [4]. An additional challenge is the need for the safe collection and handling of specimens to prevent infection of the medical staff. The lack of high-containment laboratories in the areas where the need is greatest has led to limited Lassa fever assay development and validation studies [5].

While viral culture has been the “gold standard” for the diagnosis of Lassa fever, nucleic acid detection methods have become the mainstay approach for many laboratory-based diagnostics. This type of assay can have unparalleled sensitivity and specificity. While costs of nucleic acid-based testing were initially high, automation and miniaturization have been bringing costs down each year. However, probe design is challenging due to the high sequence variability of the various strains of LASV [5].

Alternatively, antigen and antibody tests have been developed for LASV, which are attractive given the cost constraints and the austere conditions where these tests are most often required [6,7,8]. Both antigen and antibody tests have their strengths and weaknesses, as their ability to adequately identify virus is dependent on the number of days which have elapsed between infection and sample collection. Thus, for adequate identification, it will likely be necessary to develop multiplexed or parallel assays where a number of targets are assayed for simultaneously. Most antigen tests are based on the detection of LASV nucleoprotein (NP). NP antigen is detectable for the first week following infection with Lassa fever, but wanes during the second week as the levels of immunoglobulins increase [9]. Fatal cases of Lassa fever exhibited higher levels of NP versus nonfatal cases, suggesting utility for monitoring the course of the infection that one day might aid in selection of treatments [10]. Since NP is present earlier during the course of infection, it yields an earlier diagnosis than antibody detection, and represents the actual presence of virus, thus providing actionable information. Antibody, on the other hand, can be present in patients long after the infection has resolved, meaning detection of antibody is not always indicative of an active infection, just as absence of NP does not rule out a persistent viremia, since NP levels become undetectable over time [8].

Currently, the antibody-based detection of NP has utilized conventional antibodies [6,11]. While effective, these reagents cannot easily be genetically manipulated and tailored for optimization with specific detection platforms. Furthermore, polyclonal antibodies can show variability in activity from lot to lot, while monoclonal antibodies are produced by cell lines that require careful maintenance to prevent loss. We have sought to augment conventional antibodies with recombinant single-domain antibodies (sdAb) derived from the immune repertoire of an immunized llama. In addition to conventional antibodies, camelids, including llamas, possess classes of heavy-chain-only antibodies where the binding is mediated by an unpaired variable heavy domain (VHH) [12]. This simplifies the creation of recombinant binding molecules as the binding requires only the single variable domain, not the pairing of the variable heavy and light domains as in conventional antibodies. Recombinantly expressed VHH are termed sdAb or nanobodies [13].

These sdAb also possess other advantages, such as being more thermally stable, having the ability to refold following thermal denaturation, and being easier to recombinantly produce than most single-chain antibodies (recombinantly derived binding domains from conventional antibodies) [14]. Herein, we describe in detail the selection and evaluation of sdAb specific for the LASV NP, which could, with continued optimization of their functionality, be of value for the detection and diagnosis of Lassa fever.

## 2. Materials and Methods

### 2.1. Reagents

Most reagents were obtained from VWR International (Radnor, PA, USA), Sigma Aldrich (St. Louis, MO, USA), or Thermo Fisher Scientific (Waltham, MA, USA). Cloning reagents were purchased from New England Biolabs (Ipswich, MA, USA). Zalgen (Germantown, MD, USA) was the source of the recombinant NPn (N-terminal portion of NP encoding residues 1-340), NPc (C-terminal portion of NP encoding residues 341–569), and Lassa virus-like particles (VLPs). All LASV reagents were based on LASV lineage IV (Josiah strain).

### 2.2. Selection and Production of sdAb

A llama was immunized with Lassa NPn (N-terminal portion of NP) under contract with Triple J Farms, Bellingham, WA. Triple J Farms has an active Institutional Animal Care and Use Committee which reviews all immunization protocols, and approved this protocol. Anticoagulated blood was shipped overnight and buffy coat cells were isolated by centrifugation using Novamed Uni-Sep tubes. RNA was isolated from which cDNA was produced, amplifying the mRNA of the variable heavy domains, from which a phage display library was constructed as described previously [15]. Panning on NPn protein adsorbed in the wells of a microtiter plate was performed as described [15]. Using both monoclonal phage enzyme-linked immunosorbent assay (ELISA) and monoclonal phage MagPlex assay, potentially positive clones were identified after rounds two and three [16].

To facilitate periplasmic production, the coding sequences for the sdAb were cloned from the pecan21 [17] phage display vector into pET22b [18]. To produce the sdAb, expression plasmids were transformed into Tuner (DE3). Cultures (50 mL in terrific broth [TB] with 100 µg/mL ampicillin) were started from freshly transformed colonies and grown in shake-flasks overnight at 25 °C. The next day, each culture was added to 450 mL of TB with ampicillin and incubated with shaking for 2 h at 25 °C. Cultures were then induced using isopropyl-D-1 thiogalactoside (0.5 mM) and incubated for another 2 h.

Purification of sdAb was through an osmotic shock protocol followed by immobilized metal affinity chromatography (IMAC) and fast protein liquid chromatography as described previously [19]. Nearly all the protein eluted from the gel filtration column as single peak consistent with the sdAb being a monomer; very few contaminants remained following IMAC chromatography. UV absorption was utilized to determine sdAb concentration, and sdAb preparations were refrigerated for immediate use or frozen for long-term storage.

### 2.3. Surface Plasmon Resonance

A ProteOn XPR36 (Bio-Rad, Hercules, CA, USA) was used to measure the binding kinetics of the sdAb. Lanes of a GLC chip were immobilized with Lassa NPn and NPc from Zalgene diluted to 20 µg/mL in 10 mM sodium acetate pH 5.0, as described previously [20]. The binding kinetics of each sdAb were determined by flowing a range of concentrations of each sdAb over the chip to produce an array of binding curves. The chip was regenerated using 0.085% phosphoric acid between data sets. Data were corrected for interspot and zero concentration. The ProteOn Manager 2.1 software was employed for analysis of the data using the Langmuir model. The standard error of the fits was less than 10%.

### 2.4. Determining Melting Temperature and Refolding by Circular Dichroism

Circular dichroism (CD) was performed using a Jasco J-815 Spectropolarimeter, following a protocol used in prior work [15]. The sdAb samples were diluted to 15 μg/mL in deionized water and placed in a quartz cuvette with 1 cm path length and CD was measured at an ultraviolet wavelength between 204 and 206 nm. The sdAb samples were heated from 25 °C to 95 °C at a rate of 2.5 °C/min and then cooled back to 25 °C at the same rate.

### 2.5. MagPlex Direct Binding and Homogeneous Sandwich Assays

Specificity and an indication of affinity were appraised via the direct binding of the sdAb to Lassa NPn recombinant protein immobilized on MagPlex magnetic microspheres (Luminex, Austin, TX, USA). The Lassa NPn along with Lassa NPc and several unrelated viral proteins were immobilized to unique sets of MagPlex microspheres using the standard immobilization protocol provided by the manufacturer.

Using a 10-fold excess of EZ-Link NHS-LC-LC-Biotin (Thermo Fisher Scientific), each sdAb was biotinylated for 30 min; excess biotin was removed using Zeba spin columns (Thermo Fisher Scientific). The absorbance at 280 nm was used to calculate the concentration of biotinylated sdAb (Bt-sdAb). Dilutions of each Bt-sdAb in PBSTB (phosphate-buffered saline [PBS] + 0.05% Tween + 0.1% bovine serum albumin) were prepared in round-bottom polypropylene microtiter plates (VWR). The mixture of antigen-coated MagPlex microspheres was added to the wells. The plate was washed and then incubated with 5 µg/mL streptavidin-conjugated phycoerythrin (SA-PE) for 30 min, washed, and binding evaluated on the MAGPIX instrument (Luminex).

Homogeneous sandwich format MagPlex bead assays were performed in order to demonstrate the ability of the sdAb to act as both the capture and recognition reagent for the detection of Lassa VLPs. For this assay, each sdAb was immobilized to a set of MagPlex microspheres as described above and each was then tested for their ability to function as a capture antibody. Initial tests evaluated all the clones as the biotinylated recognition molecule in the assay, however, as clone H6 provided the best response, the assay was repeated using only Bt-H6.

## 3. Results

**Figure 1:**
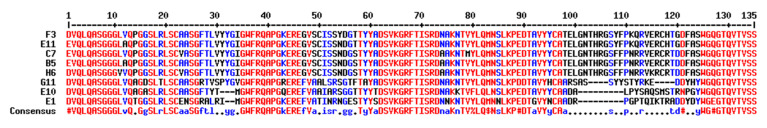
Protein sequences of the eight representative sdAb that were produced and evaluated. Sequences are presented using one-letter amino acid abbreviations. Multalin was used to generate the alignment; red denotes high homology positions while lower homology is in blue [23]. Numbering is sequential based on the F3 sequence. Using this numbering, CDR 1 is from 26 to 33, CDR 2 is from 51 to 58, and CDR3 is from 97 to 124.

After a llama was immunized with the Lassa NPn, a phage display library was generated to capture the VHH immune repertoire of the animal. Following three rounds of panning on LASV NPn recombinant protein, the evaluation of monoclonal phage from rounds two and three by MagPlex and ELISA generated 22 putative binding clones. Ten positive clones with signals at least twice the background were identified from the second round out of 96 screened, while 12 out of 96 third-round clones had signal at least four times the background and were chosen for further evaluation. Sequencing the selected prospective Lassa NPn binding sdAb revealed that the isolated clones sorted into four distinct sequence families based on homology of their CDR 3 sequence. One family was found to dominate with 16 members while the other families were represented by only one, two, or three clones. The large sequence family possessed an extra pair of cysteine residues that are presumed to form a noncanonical disulfide bond between Framework 2 and CDR 3 [21,22]; we term this the two disulfide bond (2-DSB) family. The 2-DSB family also had a relatively long CDR 3. The eight clones shown in Figure 1 represent clones from the four families that were prepared and evaluated. The G11 family had one other member, the E10 family had two other members, and E1 was the only member of its family.

Five representative sequences from the 2-DSB family, and one each from the other families, were cloned into the pET22b expression vector and produced via *E. coli* in half-liter-scale shake flasks and purified. The protein produced was virtually all monomeric with very little aggregated material observed. Table 1 shows the protein yields. For these sdAb, the presence of the additional disulfide bond appeared to impair production, as the three clones having only the single consensus disulfide bond all produced at levels of 5 mg/L or better, while the 2-DSB family ranged from a low of 0.8 to a high of 3.5 mg/L. The addition of the pTUM4 helper plasmid [24], which we have shown to aid in the production of sdAb with multiple disulfide bonds [25], provided less than two-fold improvement in protein production, suggesting that it is possible another feature of the 2-DSB family negatively impacts production.

The melting temperature (Tm) and refolding ability of each clone were measured using CD (Table 1). The 2-DSB family had Tms that ranged from 63 to 66 °C, suggesting the point mutations within the family members had little impact on Tm. The refolding ability of the 2-DSB family members, however, varied from a low of 30% to a high of 93%.

Refolding ability showed no proportionality with production potential, suggesting no linkage between refolding and production. None of the other three families that had only the single conserved disulfide bond had a Tm over 60 °C or refolded remarkably well. Thus, exploring some possible sequence modifications that could lead to enhanced Tm and refolding ability for these binders, and perhaps codon optimization of the 2-DSB family to improve production, would be useful pursuits [26].

**Figure A1:**
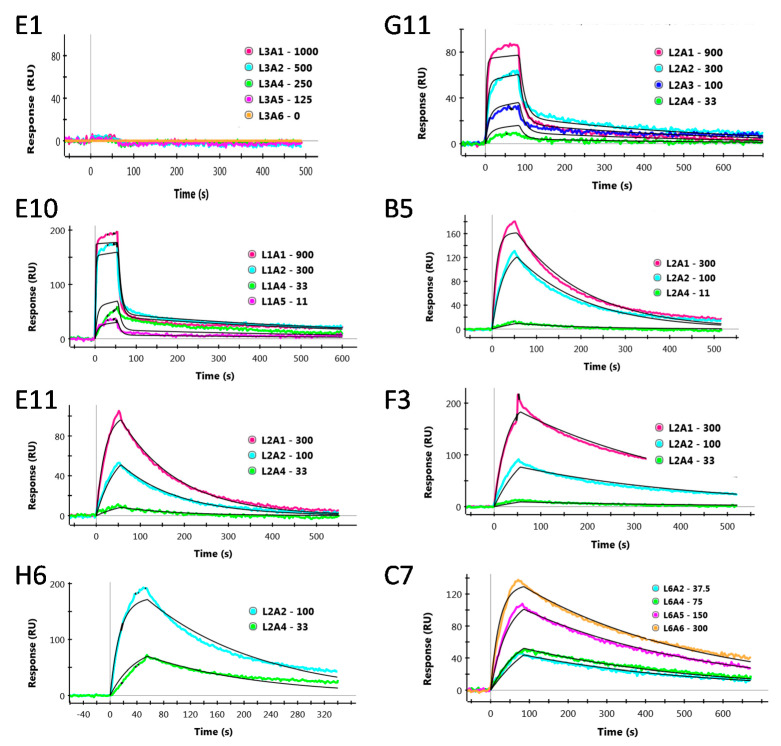
Representative surface plasmon resonance data for each of selected Lassa NP binding sdAb. The data for each clone is presented to the right of it’s name, in order from top to bottom, and left to right, clone E1, G11, E10, B5, E11, F3, H6, and C7.

The binding kinetics of the sdAb on a surface coated with Lassa NPn recombinant protein were evaluated by SPR; results are presented in Table 2 (color represents different sequence families). Characteristic SPR plots are shown in Appendix A, Figure A1. The three families with only the canonical disulfide bond showed relatively poor affinities in comparison to the 2-DSB family, ranging from a KD of 69 nM to having no binding observed at all. The 2-DSB family all have superior off rates which ranged from 7.6 to 3.0 × 10^−3^ and the KD for the family was approximately 10 nM. None of the sdAb showed any binding to NPc (not shown).

**Figure 2:**
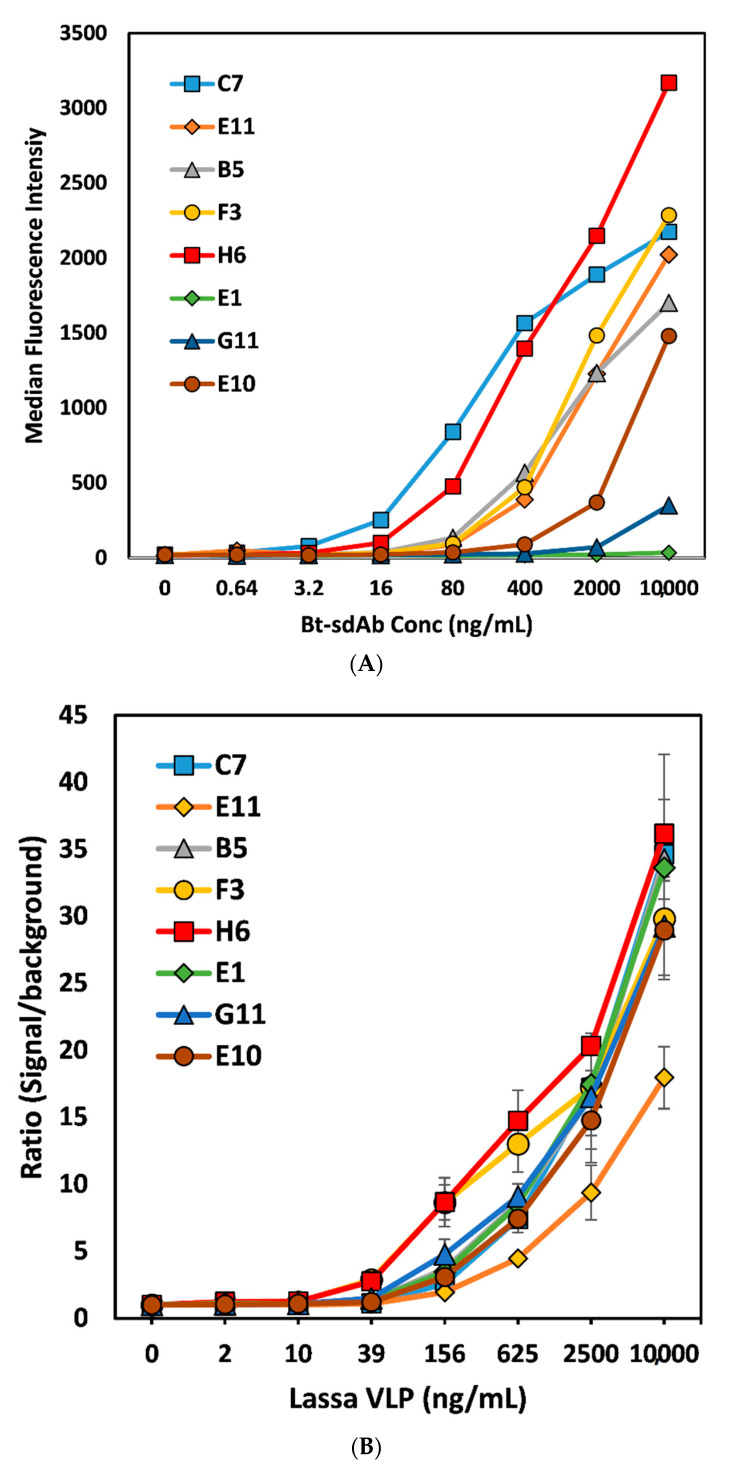
(**A**) MagPlex direct binding assay, where Lassa NPn was immobilized on MagPlex magnetic microspheres and biotinylated sdAb allowed to bind at different concentrations, followed by addition of SA-PE. (**B**) Homogeneous MagPlex sandwich fluoroimmunoassay; each sdAb was immobilized on a set of MagPlex magnetic microspheres and incubated in the presence of Lassa VLPs and biotinylated sdAb H6 and SA-PE. The amount of SA-PE bound to each microsphere set was evaluated following a single wash step. This experiment was repeated on three separate days in triplicate. Shown is the average of the three replicates from one experiment; the error bars represent the standard deviation of the mean.

The MagPlex direct binding assay of the Bt-sdAb to immobilized Lassa NPn confirmed the superiority of the 2-DSB family for their ability to bind relative to representatives of the three other families (Figure 2A). All sdAb pairs were examined for their ability to perform as both capture and recognition elements in a sandwich format. After some optimization and selection of the Bt-H6 sdAb as the best performing recognition antibody, we performed a homogeneous sandwich fluoroimmunoassay for the detection of Lassa VLPs (Figure 2B).

## 4. Discussion

This work describes the initial selection and characterization of sdAb that are specific for the Lassa NPn recombinant protein. Most of the selected binders fell into one large family where all the members possessed a second disulfide bond between framework 2 and CDR 3, typical of subfamily 3 of llama VHH [22]. As with other members of subfamily 3, the isolated 2-DSB family members possess a longer-than-average CDR3 with a length of 27 versus conventional llama sdAb whose average CDR 3 length is 14 [22]. The sdAb’s conserved disulfide bond primarily provides enhanced stability to the structure of the sdAb, while the disulfide bond between framework 2 and CDR 3 can stabilize the structure; it typically plays a more critical role in binding affinity by maintaining the secondary structure of the binding loops [27,28]. Three other sequence families were also identified which had between one and three representatives. These three other clones had only the typical disulfide bond found in variable heavy domains, however, they still had long CDR 3 regions, with lengths of 18 and 19.

The melting point and ability to refold were evaluated for each of the clones. None of them were found to be remarkably stable although clone E11 did refold over 90%. It may be of interest to better understand the impact that the minor sequence differences between members of the 2-DSB family have on their ability to refold as it varied from a low of 30% for H6 to a high of 93% for E11, while their Tm was virtually the same. If these binders are developed further, one could make additional sequence changes to increase both their Tm and ability to refold [26], thus making them more attractive for applications in austere locales where refrigeration is a challenge.

The affinity of each of these binders was also evaluated. The clone E1 did not show any binding by SPR or give a significant signal in the MagPlex direct binding assay, thus it is likely that isolation of this sequence was spurious. Notably, E1 was the only member of its sequence family, increasing the likelihood that it is not a true Lassa NPn binder. Of the other clones, it quickly became clear that the 2-DSB family of binders were superior to the other two. Their affinities were in the range of 10 nM and all had off rates that were at least fivefold lower than any of the other binders examined. Nonetheless, these affinities are not as good as those frequently obtained from llama immune phage-display libraries, where frequently sub-nM KD binders are isolated. This limited affinity is borne out by the need to perform a homogenous sandwich assay, whereby limiting the dissociation time of the immune-complex, we were able to demonstrate that these binders show potential for the detection of LASV via their ability to detect Lassa VLPs that included NP in addition to glycoprotein and matrix protein.

After testing a range of conditions, we were able to demonstrate detection of Lassa VLPs down to as little as 39 ng/mL, with both the H6 and F3 capture sets having a ratio of signal/background over 3, where the limit of detection cut-off ratio is 2, a ratio we have shown previously provides a statistically significant increase [16]. While this demonstrates their potential, it is highly likely that binders with a higher affinity will be required to obtain adequate sensitivities to reliably detect for LASV infection. To achieve this goal, the two best options are to immunize another animal in an effort to obtain an immune repertoire that possesses higher affinity binders or to take advantage of avidity by preparing sdAb multimers of our current binders. In other work we, as well as others, have made sdAb multimers either via direct genetic linkage [29,30,31] or via a fusion protein that forms a homodimer such as alkaline phosphatase or rhizavidin [32,33] or fusion with a peptide that promotes multimerization [34]. These dimers and multimers have been shown to dramatically enhance both affinity and utility in immunoassays [35,36], however, it remains to be determined if such an approach would prove successful here.

The sdAb were selected on a recombinantly expressed n-terminal fragment of NP containing amino acids 1-304 of the protein from the lineage IV strain Josiah. A comparison of NP sequences from different LASV strains showed that there can be up to 12% variation in the amino acid sequence [4]. Future work should include determining if the selected sdAb are able to recognize NP from other LASV strains. Alternatively, one could do additional rounds of selection with a different LASV strain to select binders capable of binding to a common epitope. Nonetheless, the sdAb we isolated could be used in conjunction with sdAb reagents selected on NP from a variety of strains to minimize false negatives. Future studies involve evaluating the ability of the sdAb to recognize VLPs that include NP derived from other LASV lineages, and eventually their ability to detect live virus. Ultimately, one will want to compare sdAb reagents head-to-head with conventional monoclonal antibodies to directly evaluate any perceived advantage obtained and perhaps whether an assay format that includes both reagents is warranted. However, from our experience, until the sdAb have been further optimized to enhance their functionality, such a comparison would be premature. In conclusion, we have described and evaluated four families of Lassa NPn binding sdAb and hypothesize that with continued development, they may become useful reagents in the fight against Lassa fever.

## Figures and Tables

**Table 1:** Production, CD melting point and refolding, and biophysical properties.

sdAb	Yield (mg/L)	Tm (°C)	% Refold	pI	#SS Bonds
E1	8.4	59	56	7.18	1
G11	5.2	60	30	8.01	1
E10	16.4	48	15	8.64	1
E11	0.8	64	93	7.95	2
B5	1.4	63	52	7.96	2
H6	3.5	64	30	8.74	2
F3	2.7	65	59	7.89	2
C7	1.9	66	83	7.96	2

**Table 2:** Kinetic parameters of Lassa NPn binding sdAb.

Clone	ka (1/Ms)	kd (1/s)	KD (nM)
E1	-	-	NBO *
G11	3.8 × 10^5^	7.0 × 10^−2^	184
E10	1.9 × 10^6^	1.3 × 10^−1^	69
E11	1.5 × 10^5^	7.6 × 10^−3^	57
B5	3.2 × 10^5^	6.1 × 10^−3^	19
H6	6.1 × 10^5^	5.7 × 10^−3^	9.3
F3	4.0 × 10^5^	3.0 × 10^−3^	7.5
C7	5.1 × 10^5^	3.8 × 10^−3^	7.5

* NBO: no binding observed.

## References

[1] Yun N.E., Walker D.H. (2012). Pathogenesis of Lassa fever. Viruses.

[2] Branco L.M., Boisen M.L., Andersen K.G., Grove J.N., Moses L.M., Muncy I.J., Henderson L.A., Schieffellin J.S., Robinson J.E., Bangura J.J. (2011). Lassa hemorrhagic fever in a late term pregnancy from northern sierra leone with a positive maternal outcome: Case report. Virol. J..

[3] Price M.E., Fisher-Hoch S.P., Craven R.B., McCormick J.B. (1988). A prospective study of maternal and fetal outcome in acute Lassa fever infection during pregnancy. BMJ.

[4] Bowen M.D., Rollin P.E., Ksiazek T.G., Hustad H.L., Bausch D.G., Demby A.H., Bajani M.D., Peters C.J., Nichol S.T. (2000). Genetic Diversity among Lassa Virus Strains. J. Virol..

[5] Raabe V., Koehler J. (2017). Laboratory Diagnosis of Lassa Fever. J. Clin. Microbiol..

[6] Boisen M.L., Hartnett J.N., Shaffer J.G., Goba A., Momoh M., Sandi J.D., Fullah M., Nelson D.K.S., Bush D.J., Rowland M.M. (2018). Field validation of recombinant antigen immunoassays for diagnosis of Lassa fever. Sci. Rep..

[7] Boisen M.L., Uyigue E., Aiyepada J., Siddle K.J., Oestereich L., Nelson D.K.S., Bush D.J., Rowland M.M., Heinrich M.L., Eromon P. (2020). Field evaluation of a Pan-Lassa rapid diagnostic test during the 2018 Nigerian Lassa fever outbreak. Sci. Rep..

[8] Jahrling P., Niklasson B., McCormick J. (1985). Early Diagnosis of Human Lassa Fever by ELISA Detection of Antigen and Antibody. Lancet.

[9] Bausch D.G., Rollin P.E., Demby A.H., Coulibaly M., Kanu J., Conteh A.S., Wagoner K.D., McMullan L.K., Bowen M.D., Peters C.J. (2000). Diagnosis and clinical virology of Lassa fever as evaluated by enzyme-linked immunosorbent assay, indirect fluorescent-antibody test, and virus isolation. J. Clin. Microbiol..

[10] Branco L.M., Grove J.N., Boisen M.L., Shaffer J.G., Goba A., Fullah M., Momoh M., Grant D.S., Garry R.F. (2011). Emerging trends in Lassa fever: Redefining the role of immunoglobulin M and inflammation in diagnosing acute infection. Virol. J..

[11] Grove J.N., Branco L.M., Boisen M.L., Muncy I.J., Henderson L.A., Schieffellin J.S., Robinson J.E., Bangura J.J., Fonnie M., Schoepp R.J. (2011). Capacity building permitting comprehensive monitoring of a severe case of Lassa hemorrhagic fever in Sierra Leone with a positive outcome: Case report. Virol. J..

[12] Hamers-Casterman C., Atarhouch T., Muyldermans S., Robinson G., Hamers C., Songa E.B., Bendahman N., Hamers R. (1993). Naturally occurring antibodies devoid of light chains. Nature.

[13] Muyldermans S. (2013). Nanobodies: Natural Single-Domain Antibodies. Annu. Rev. Biochem..

[14] de Marco A. (2020). Recombinant expression of nanobodies and nanobody-derived immunoreagents. Protein Expr. Purif..

[15] Liu J.L., Shriver-Lake L.C., Anderson G.P., Zabetakis D., Goldman E.R. (2017). Selection, characterization, and thermal stabilization of llama single domain antibodies towards Ebola virus glycoprotein. Microb. Cell Fact..

[16] Shriver-Lake L.C., Liu J.L., Zabetakis D., Sugiharto V.A., Lee C.-R., Defang G.N., Wu S.-J.L., Anderson G.P., Goldman E.R. (2018). Selection and Characterization of Anti-Dengue NS1 Single Domain Antibodies. Sci. Rep..

[17] Goldman E.R., Anderson G.P., Liu J.L., Delehanty J.B., Sherwood L.J., Osborn L.E., Cummins L.B., Hayhurst A. (2006). Facile generation of heat-stable antiviral and antitoxin single domain antibodies from a semisynthetic llama library. Anal. Chem..

[18] Walper S.A., Liu J.L., Zabetakis D., Anderson G.P., Goldman E.R. (2014). Development and Evaluation of Single Domain Antibodies for Vaccinia and the L1 Antigen. PLoS ONE.

[19] Shriver-Lake L.C., Zabetakis D., Goldman E.R., Anderson G.P. (2017). Evaluation of anti-botulinum neurotoxin single domain antibodies with additional optimization for improved production and stability. Toxicon.

[20] Walper S.A., Lee P.A.B., Goldman E.R., Anderson G.P. (2013). Comparison of single domain antibody immobilization strategies evaluated by surface plasmon resonance. J. Immunol. Methods.

[21] Muyldermans S., Atarhouch T., Saldanha J., Barbosa J.A., Hamers R. (1994). Sequence and structure of VH domain from naturally occurring camel heavy chain immunoglobulins lacking light chains. Protein Eng..

[22] Harmsen M.M., Ruuls R.C., Nijman I.J., Niewold T.A., Frenken L.G.J., de Geus B. (2000). Llama heavy-chain V regions consist of at least four distinct subfamilies revealing novel sequence features. Mol. Immunol..

[23] Corpet F. (1988). Multiple sequence alignment with hierarchical-clustering. Nucleic Acids Res..

[24] Schlapschy M., Grimm S., Skerra A. (2006). A system for concomitant overexpression of four periplasmic folding catalysts to improve secretory protein production in Escherichia coli. Protein Eng. Des. Sel..

[25] Shriver-Lake L.C., Goldman E.R., Zabetakis D., Anderson G.P. (2017). Improved production of single domain antibodies with two disulfide bonds by co-expression of chaperone proteins in the Escherichia coli periplasm. J. Immunol. Methods.

[26] Goldman E.R., Liu J.L., Zabetakis D., Anderson G.P. (2017). Enhancing Stability of Camelid and Shark Single Domain Antibodies: An Overview. Front. Immunol..

[27] Hagihara Y., Mine S., Uegaki K. (2007). Stabilization of an immunoglobulin fold domain by an engineered disulfide bond at the buried hydrophobic region. J. Biol. Chem..

[28] Govaert J., Pellis M., Deschacht N., Vincke C., Conrath K., Muyldermans S., Saerens D. (2012). Dual Beneficial Effect of Interloop Disulfide Bond for Single Domain Antibody Fragments. J. Biol. Chem..

[29] Goldman E.R., Broussard A., Anderson G.P., Liu J.L. (2017). Bglbrick strategy for the construction of single domain antibody fusions. Heliyon.

[30] Krasniqi A., Bialkowska M., Xavier C., Van der Jeught K., Muyldermans S., Devoogdt N., D’Huyvetter M. (2018). Pharmacokinetics of radiolabeled dimeric sdAbs constructs targeting human CD20. New Biotechnol..

[31] Conrath K.E., Lauwereys M., Wyns L., Muyldermans S. (2001). Camel single-domain antibodies as modular building units in bispecific and bivalent antibody constructs. J. Biol. Chem..

[32] Liu J.L., Zabetakis D., Brozozog Lee P.A., Goldman E.R., Anderson G.P. (2013). Single Domain Antibody Alkaline Phosphatase Fusion Proteins for Antigen Detection—Analysis of Affinity and Thermal Stability of Single Domain Antibody. J. Immunol. Methods.

[33] Liu J.L., Zabetakis D., Walper S.A., Goldman E.R., Anderson G.P. (2014). Bioconjugates of rhizavidin with single domain antibodies as bifunctional immunoreagents. J. Immunol. Methods.

[34] Zhang J., Tanha J., Hirama T., Khieu N.H., To R., Tong-Sevinc H., Stone E., Brisson J.-R., MacKenzie C.R. (2004). Pentamerization of Single-domain Antibodies from Phage Libraries: A Novel Strategy for the Rapid Generation of High-avidity Antibody Reagents. J. Mol. Biol..

[35] Swain M.D., Anderson G.P., Serrano-Gonzalez J., Liu J.L., Zabetakis D., Goldman E.R. (2011). Immunodiagnostic reagents using llama single domain antibody-alkaline phosphatase fusion proteins. Anal. Biochem..

[36] Wang S., Zheng C., Liu Y., Zheng H., Wang Z. (2008). Construction of multiform scFv antibodies using linker peptide. J. Genet. Genom..

